# Pleomorphic Lung Carcinoma Response to Treatment With BRAF and MEK Inhibitors: A Case Report

**DOI:** 10.7759/cureus.79073

**Published:** 2025-02-15

**Authors:** Hideki Endoh, Satoshi Wasamoto

**Affiliations:** 1 Department of Thoracic Surgery, Saku Central Hospital Advanced Care Center, Saku, JPN; 2 Department of Respiratory Medicine, Saku Central Hospital Advanced Care Center, Saku, JPN

**Keywords:** braf mutations, dabrafenib, lung cancer, non-small cell lung, pleomorphic carcinoma, trametinib

## Abstract

Pleomorphic carcinoma is one of the most difficult non-small cell lung cancers to treat with cytotoxic agents. Early recurrence after pulmonary resection is common, and the prognosis is poor. Herein, we report a case of pleomorphic carcinoma effectively treated via molecular targeting. An 80-year-old man relapsed within six months of undergoing left lower lobectomy for pathological stage IIB pleomorphic carcinoma. The initial treatment consisted of carboplatin, pemetrexed, and pembrolizumab, together with opioids (for severe pain due to pleuritis carcinomatosis and bone metastasis) and home oxygen therapy. Following the detection of a BRAF mutation (V600E) via whole-genome sequencing, the treatment was switched to dabrafenib (a BRAF inhibitor) and trametinib (a MEK inhibitor). One month later, the previously observed pleural effusion disappeared, and the radiological pulmonary findings were normal. The patient’s pain diminished, the opioid dose was reduced, and home oxygen therapy was discontinued. This condition was maintained for approximately nine months; however, the patient died 11 months after treatment. Although controlling pulmonary pleomorphic carcinoma is challenging, the present case illustrates the effectiveness of BRAF and MEK inhibitors in cases with BRAF mutations, even those involving octogenarians.

## Introduction

Pleomorphic carcinoma is a rare subtype of non-small cell lung cancer (NSCLC) that contains ≥10% spindle or giant cells. It has an incidence of 0.1-0.4%, an aggressive clinical course, and a poor prognosis [1,2]. Because of its rarity, no definitive treatment options have yet been established.

In NSCLC, somatic mutations of driver genes have been identified based on examination of surgical and biopsy specimens. These genes include BRAF, which encodes the serine-threonine kinase BRAF, as well as *EGFR*, *ALK*, *ROS1*, *MET*, *PIK3CA*, *RET*, *KRAS*, and *HER2*.

BRAF mutations have been found in 2-5% of NSCLC in Western populations and 0.5-2% of NSCLC in East Asian populations [3]. The most common BRAF mutation, V600E, is an oncogenic driver of NSCLC, and targeted therapy with a combination of dabrafenib and trametinib has been approved.

Here, we report a case of pleomorphic carcinoma with a BRAF mutation that was effectively treated with dabrafenib and trametinib.

## Case presentation

**Figure 1:**
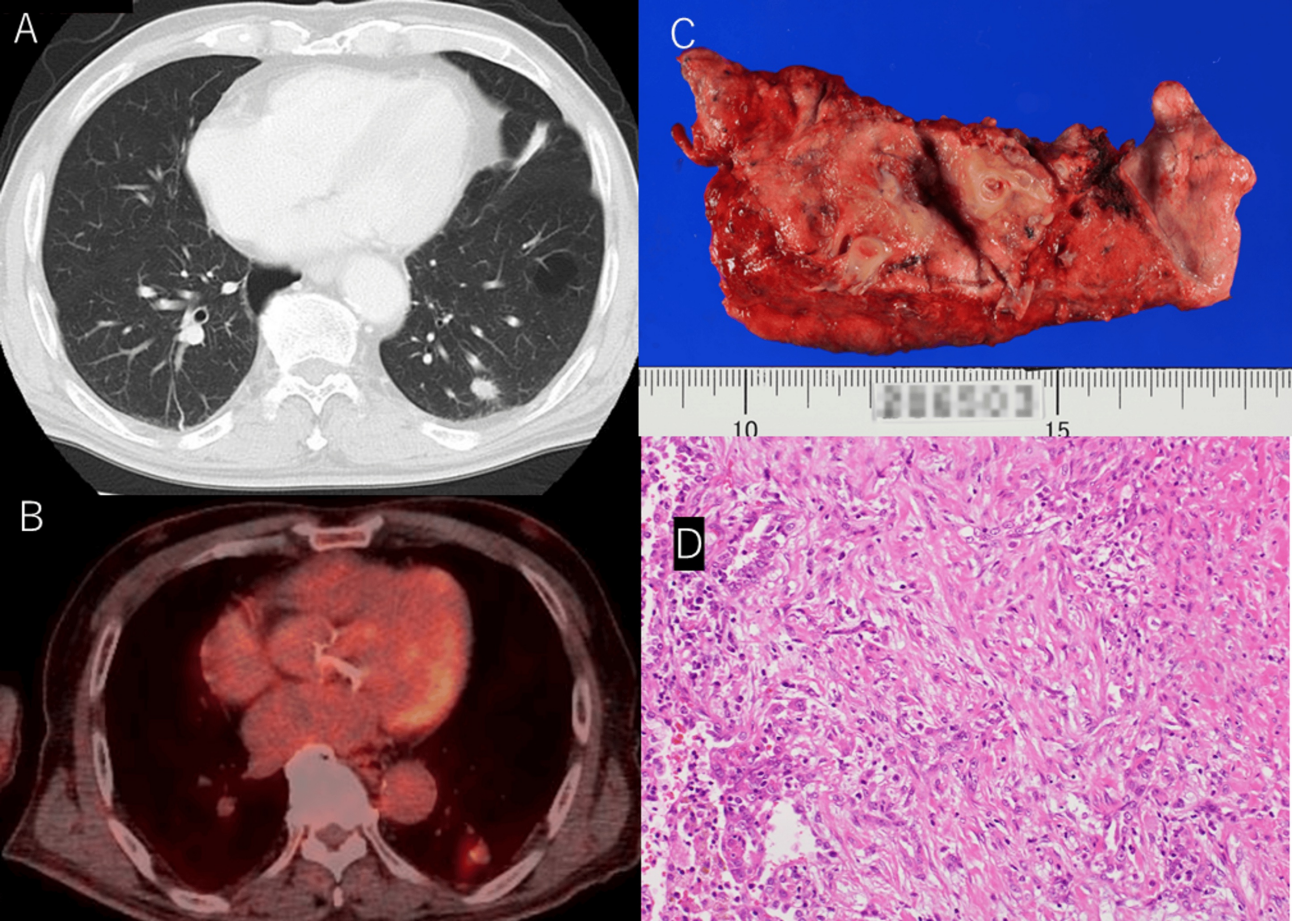
Imaging and histological findings A: A small nodule on chest computed tomography. B: Uptake of fluorodeoxyglucose on positron emission tomography. C: Partial resection of the tumor in the left lower lobe. D: Adenocarcinoma with spindle cells (30%) on microscopy (hematoxylin and eosin staining, 20x). The neoplastic cells are negative for S100, HMB 45, CD34, CD31, and CAM 5.2.

An 80-year-old man presented to our hospital after medical examination in another hospital without complaints. A small solid mass was identified at the peripheral side of the left lower lobe (S10) on chest computed tomography and the uptake of fluorodeoxyglucose was observed using positron emission tomography (Figures 1A, 1B). He was diagnosed with clinical stage IA2 lung cancer and underwent left lower lobectomy with lower mediastinal and hilar lymph node dissection for clinical stage IA2 NSCLC at Saku Central Hospital Advanced Care Center. The patient was an ex-smoker (60 pack-years), and the tumor diameter was 1.3 cm. The pathological diagnosis was pleomorphic carcinoma with pulmonary metastasis (T3N0M0) (Figures 1C, 1D). Although the pathological stage was IIB, the patient was administered oral tegafur-uracil as adjuvant chemotherapy.

**Figure 2:**
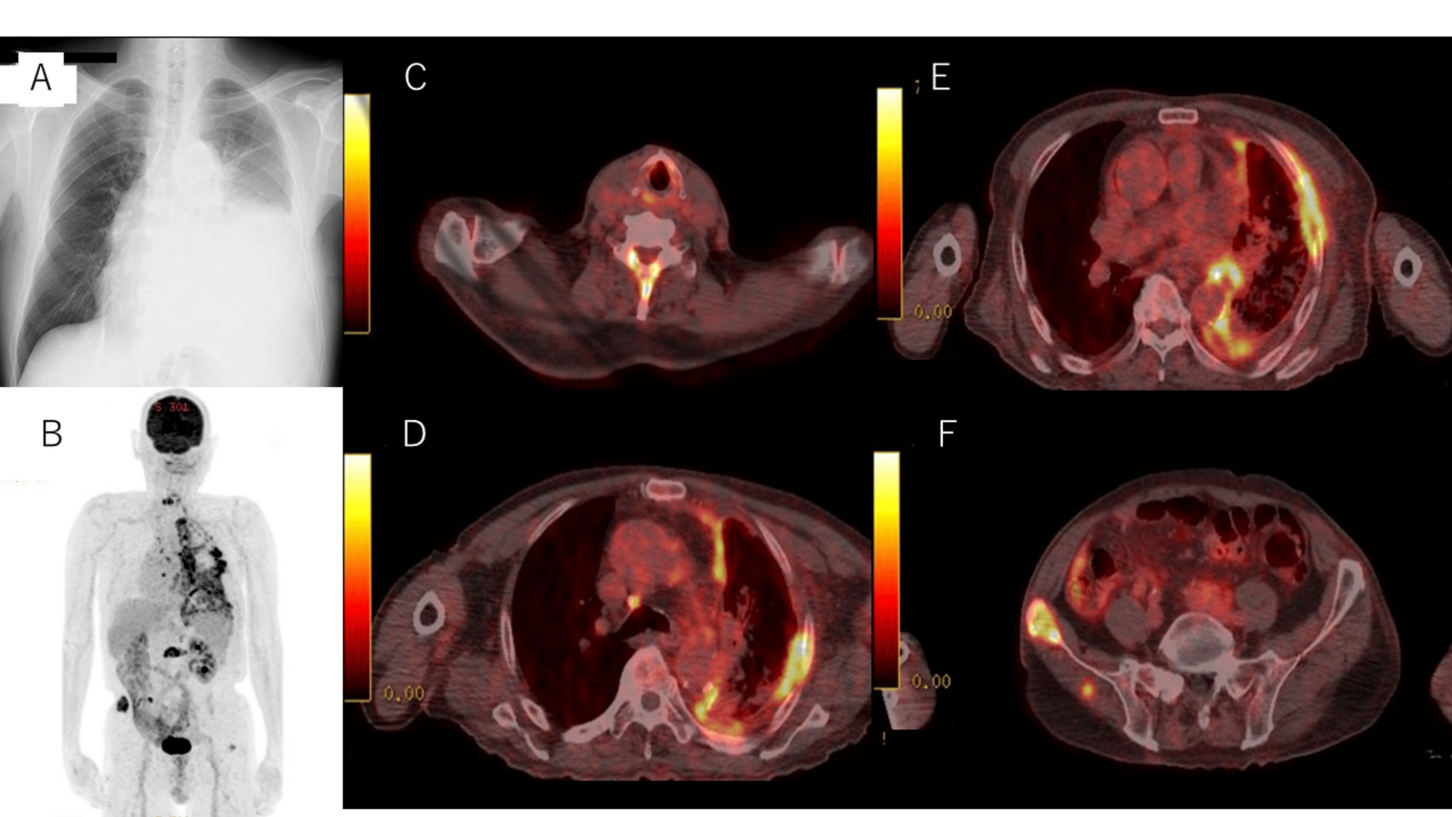
Imaging at recurrence A:  Left pleural effusion on chest radiography. B-F: Fluorodeoxyglucose positron emission tomography shows pleural dissemination and multiple bone metastases.

Approximately four months after surgery, left pleural effusion developed; two cytologic examinations revealed no malignant cells. Drainage and talc levels were adjusted several times to control the effusion; however, the patient experienced severe pain. Fluorodeoxyglucose positron emission tomography revealed pleural carcinomatosis and multiple bone metastases (Figures 2A-2F) which was diagnosed as recurrent stage IV lung cancer. The patient had chest pain and difficulty breathing; therefore, he began home oxygen therapy (2-L/min) and took 40 mg/day of oxycodone.

**Figure 3:**
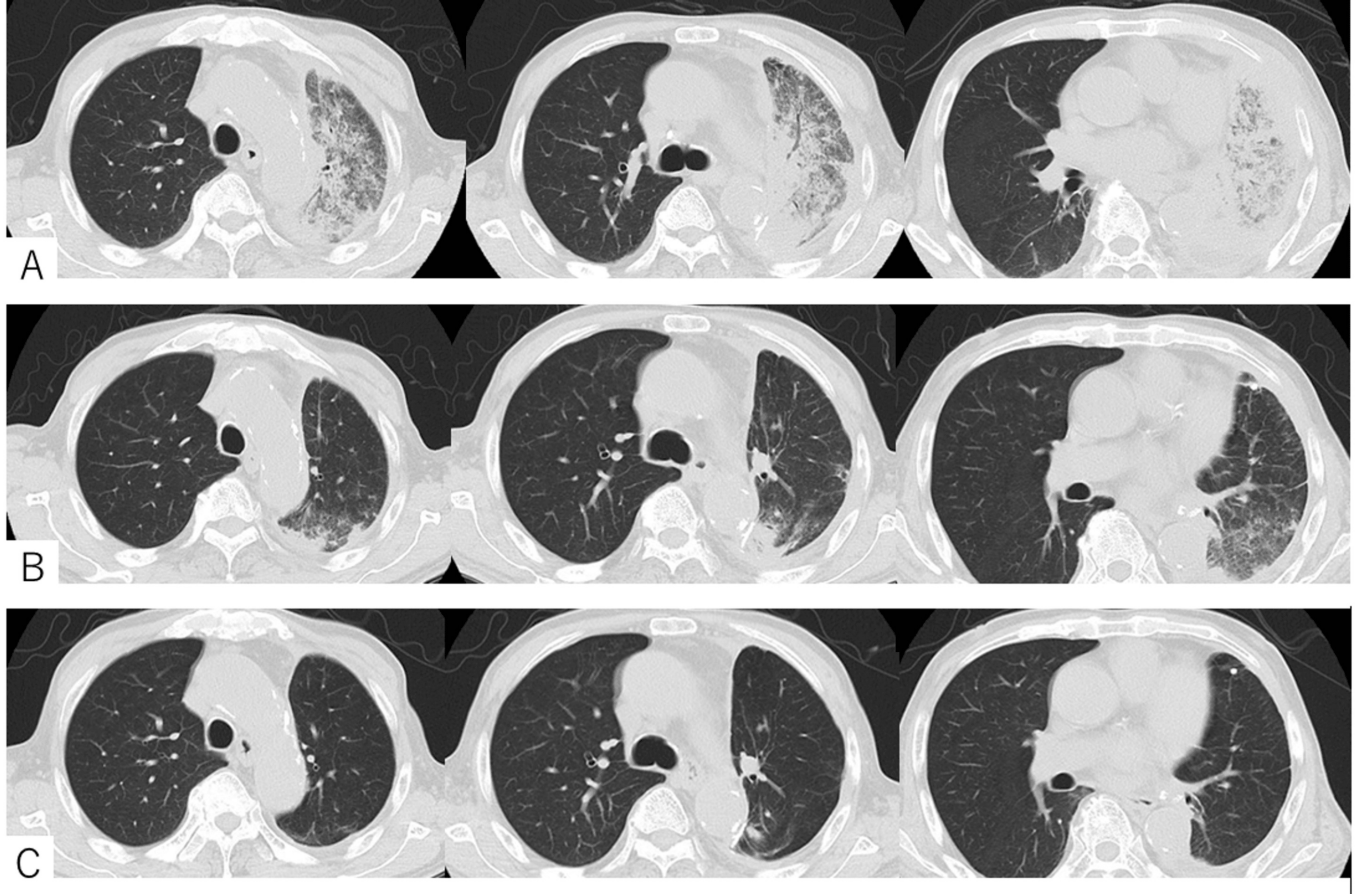
Clinical course after treatment with a BRAF inhibitor A:  Chest computed tomography at the start of treatment. B:  Five weeks after treatment. C:  Status at three months after treatment was maintained until six months after treatment.

A combination of carboplatin, pemetrexed, and pembrolizumab was administered as the initial treatment because this regimen is the recommended first-line treatment for non-squamous cancers with no or unknown biomarkers at the time of treatment. Following the detection of the V600E mutations via whole-genome sequencing, the treatment was switched to dabrafenib and trametinib, which are BRAF and MEK inhibitors, respectively. One month later, the pleural effusion lessened, and the radiological pulmonary findings were normal (Figures 3A-3C). The patient’s pain diminished, the opioid dose was reduced, and home oxygen therapy was discontinued. This condition was maintained for approximately nine months; however, the patient died 11 months after treatment. All clinical procedures were performed in the same hospital.

## Discussion

There are three types of BRAF mutations. V600 mutations activate normally inactive BRAF monomers (class I), kinase-activating non-V600 mutations allow BRAF to function as a RAS-independent dimer (class II), and kinase-impaired non-V600 mutations amplify MEK signaling in the presence of activated upstream receptor tyrosine kinases or co-alterations that increase RAS activity (class III). In a previous study of 237 patients with NSCLC who have BRAF mutations, 107 (45%) had class I mutations [4]. Class I BRAF mutation includes V600E/K/D and directly activates downstream MEK1/2 in an RAS-independent manner, resulting in deregulated proliferation and shortened survival times. By preventing downstream signaling, BRAF inhibitors (dabrafenib, trametinib, and encorafenib) in combination with MEK inhibitors (trametinib, cobimetinib, and binimetinib) inhibit proliferation and increase survival times [5].

Handa et al. analyzed 44 cases of pulmonary pleomorphic carcinoma [6]. The prognosis of pulmonary pleomorphic carcinoma was poor, with one-year overall and recurrence-free survival rates of 52.6% and 45.8%, respectively, even though 26 (59.1%) cases were clinical stage I and none were stage IV.

Among the 55 pleomorphic carcinomas examined by Kojima et al., pathogenic mutations in *PIK3CA* and *EGFR *were found in four (7%) and five (9%) cases, respectively [7]. A previous report identified osimertinib as an effective treatment for pleomorphic carcinomas harboring *EGFR* mutations with exon 19 deletions [8]. However, further studies are required to validate the effectiveness of osimertinib and other epidermal growth factor-tyrosine kinase inhibitors in pleomorphic carcinoma [8]. Pleomorphic carcinoma has been associated with the high expression of programmed death-ligand 1 [9,10]. The effectiveness of immune checkpoint inhibitors against pulmonary pleomorphic carcinoma has been reported [11].

Most histological subtypes with *MET* exon 14 skipping are adenocarcinomas (10/15, 66.7%), followed by pleomorphic carcinomas (3/15, 20.0%) [12]. Management of pleomorphic carcinomas with *ROS1* [13] and *ALK* [14] rearrangements using lorlatinib and crizotinib, respectively, has been reported.

Pulmonary pleomorphic carcinomas contain adenocarcinoma or squamous and spindle cell components. The epithelial components are adenocarcinoma (63.6%) and squamous cell carcinoma (36.4%). Kaira et al. reported that *EGFR* mutations were found in the adenocarcinomatous component but not in the sarcomatoid component [2]. BRAF mutations may also occur in only adenocarcinomas; thus, BRAF inhibitors may not appreciably affect spindle cell carcinomas [15]. Dabrafenib plus trametinib is the standard treatment for patients with mutant metastatic NSCLC with BRAF V600E mutations; in a phase II trial, the median progression-free survival time was 14.6 months in treatment-naïve patients [16]. In patients who are 80 years or older with NSCLC harboring *EGFR* mutations or *ALK* translocations, the average survival time was 13.6 months [17]. In the present case, the progression-free survival was relatively short (approximately nine months). Although limited evidence supports the medical advantages of using immune checkpoint inhibitors for older patients with advanced NSCLCs, these inhibitors were continuously administered to a 78-year-old man with pleomorphic carcinoma for eight months [18]. Therefore, immune checkpoint inhibitors could be a treatment option for such cases.

## Conclusions

Herein, we report the case of a pulmonary pleomorphic carcinoma harboring a BRAF mutation. Pulmonary pleomorphic carcinoma is one of the most difficult lung cancers to control using anticancer drugs. However, molecular-targeted drugs can mitigate the patient’s distress for more than half a year. This clinical course might not be always promising, and careful management might be needed for older patients than for younger patients. The present case showcases genomic mutations and the use of BRAF and MEK inhibitors in patients with pleomorphic carcinoma, including octogenarians.

## References

[1] Chang YL, Lee YC, Shih JY, Wu CT (2001). Pulmonary pleomorphic (spindle) cell carcinoma: peculiar clinocopathologic manifestations different from ordinary non-small cell carcinoma. Lung Cancer.

[2] Kaira K, Horie Y, Ayabe E (2010). Pulmonary pleomorphic carcinoma: a clinicopathological study including EGFR mutation analysis. J Thorac Oncol.

[3] Sakai T, Matsumoto S, Ueda Y (2023). Clinicogenomic features and targetable mutations in NSCLCs harboring BRAF non-V600E mutations: a multi-institutional genomic screening study (LC-SCRUM-Asia). J Thorac Oncol.

[4] Dagogo-Jack I, Martinez P, Yeap BY (2019). Impact of BRAF mutation class on disease characteristics and clinical outcomes in BRAF-mutant lung cancer. Clin Cancer Res.

[5] Sforza V, Palumbo G, Cascetta P (2022). BRAF inhibitors in non-small cell lung cancer. Cancers (Basel).

[6] Handa Y, Ikeda T, Hanaki H, Miyata Y, Yoshimura K, Okada M, Mukaida H (2023). Pulmonary pleomorphic carcinoma: its clinical behavior, prognostic factor, and keys to treatment. JJLC.

[7] Kojima K, Imai S, Samejima H, Fujiwara A, Tokunaga T, Yoon H, Okishio K (2022). PIK3CA mutations associated with a poor postoperative prognosis in patients with pulmonary pleomorphic carcinoma: a retrospective cohort study. BMC Cancer.

[8] Kano Y, Kataoka N, Kunimatsu Y (2022). Pulmonary pleomorphic carcinoma harboring EGFR mutation successfully treated with osimertinib: a case report. Medicina (Kaunas).

[9] Kaira K, Shimizu K, Endoh H (2020). Prognostic significance of tumor immunity in surgically resected pulmonary pleomorphic carcinoma. Anticancer Res.

[10] Naito M, Tamiya A, Takeda M (2019). A high PD-L1 expression in pulmonary pleomorphic carcinoma correlates with parietal-pleural invasion and might predict a poor prognosis. Intern Med.

[11] Miyashita K, Karayama M, Inoue Y (2021). Efficacy of immune checkpoint inhibitors in non-small cell lung cancer with uncommon histology: a propensity-score-matched analysis. BMC Pulm Med.

[12] Watari N, Yamaguchi K, Terada H (2022). Characteristic computed tomography features in mesenchymal-epithelial transition exon14 skipping-positive non-small cell lung cancer. BMC Pulm Med.

[13] Wu CW, Yang CY, Chang YL, Shih JY (2020). Successful management of a ROS1-rearranged pulmonary pleomorphic carcinoma using serial tyrosine kinase inhibitors. Onco Targets Ther.

[14] Lin L, Huang F, Chen F, He Y, Hu J, Cao X (2018). Anaplastic lymphoma kinase (ALK)-rearranged pulmonary pleomorphic carcinoma successfully treated with crizotinib. J Int Med Res.

[15] Wei Y, Wang L, Jin Z, Jia Q, Brcic L, Akaba T, Chu Q (2024). Biological characteristics and clinical treatment of pulmonary sarcomatoid carcinoma: a narrative review. Transl Lung Cancer Res.

[16] Planchard D, Smit EF, Groen HJM (2017). Dabrafenib plus trametinib in patients with previously untreated BRAF(V600E)-mutant metastatic non-small-cell lung cancer: an open-label, phase 2 trial. Lancet Oncol.

[17] Tufman A, Kahnert K, Duell T (2017). Frequency and clinical relevance of EGFR mutations and EML4-ALK translocations in octogenarians with non-small cell lung cancer. Onco Targets Ther.

[18] Yamasaki M, Matsumoto Y, Nakamoto K, Hattori N (2021). Pulmonary pleomorphic carcinoma in an elderly patient treated with pembrolizumab shows a marked response. J Cancer Res Ther.

